# Trophic niche segregation in a guild of top predators within the Mediterranean Basin

**DOI:** 10.1093/cz/zoae001

**Published:** 2024-01-30

**Authors:** Samuele Ramellini, Emanuele Crepet, Stefano Lapadula, Andrea Romano

**Affiliations:** Department of Environmental Science and Policy, University of Milan, Milan, Italy; Department of Environmental Science and Policy, University of Milan, Milan, Italy; Department of Environmental Science and Policy, University of Milan, Milan, Italy; Department of Environmental Science and Policy, University of Milan, Milan, Italy

**Keywords:** diet, foraging guild, interspecific competition, Mediterranean Basin, sympatry, trophic niche partitioning

## Abstract

Niche theory predicts that closely related and ecologically similar species with overlapping distribution ranges can coexist through resource partitioning that limits interspecific competition. However, studies examining the mechanisms promoting coexistence of top predators at a large geographical scale are still scant. Here, we describe the foraging ecology of 3 sympatric owl species (Northern long-eared owl [*Asio otus*], Tawny owl [*Strix aluco*], Eurasian eagle owl [*Bubo bubo*]) in the Mediterranean Basin. We review 160 studies reporting diet information (212,236 vertebrate preys) and investigate among-species differences in diet metrics (diversity, evenness, prey size, and proportion of mammals) and their variation along geographical and environmental gradients. Moreover, we test whether diet metrics differ in presence or absence of the other predators. All the 3 species mainly rely on small mammals, but they significantly differ in diet metrics. The smallest predator (i.e., long-eared owl) shows a higher level of specialism on small mammals (highest proportion but lowest diversity of mammals in the diet) compared to the larger ones. In addition, mean prey size significantly increases with predator body size (long-eared owl < tawny owl < eagle owl). Finally, interspecific competition results in an increase of diet diversity and evenness in the long-eared owl, and species’ diet also varies in response to environmental factors. The 3 species thus segregate along several dietary niche axes over a large spatial scale and according to both morphological characteristics (i.e., body size) and environmental variables. Such dietary niche segregation may adaptively buffer interspecific competition costs, ultimately allowing coexistence.

Niche partitioning is considered one of the main mechanisms favoring and maintaining the sympatric coexistence of species with similar morphological and ecological features (MacArthur 1958; Schoener 1974). Many species partition their niches differentiating along the trophic niche axes. This is especially the case whenever a group of species share similar trophic resources and foraging strategies, the so-called foraging guilds (Root 1967). In foraging guilds, interspecific competition may become disproportionately high, and among-species dietary differentiation may thus ease coexistence (see e.g., Garcia and Arroyo 2005; Agarwal and Rastogi 2009). Trophic niche partitioning has been frequently described in animals and can be the result of several processes (Pianka 1973). Among these, spatial (Navarro et al. 2009) and/or temporal partitioning during foraging (Lear et al. 2021), as well as differences in hunting strategies (Michalko and Pekár 2016) have been observed in several taxa. Another mechanism that can prevent competitive exclusion is ecological character displacement, which leads to morphological differentiation among species and allows the exploitation of slightly different resources (Nakano et al. 2020). As a consequence, resource partitioning may be primary, whenever competitors do not overlap in their main resource, thus resulting in trophic segregation (Macarthur and Levins 1967; Rosenzweig 1981; Devictor et al. 2010) or secondary, whenever competitors share their principal resource while partitioning the less valuable ones (Rosenzweig and Abramsky 1986; Gurd 2008).

Despite the large literature available on niche partitioning, uncertainty still exists about the biological and environmental conditions promoting species coexistence under high interspecific competition (Chesson and Huntly 1997; Gurevitch et al. 2000; Chase et al. 2002). This is especially the case for predators, as resource partitioning processes may play a crucial role in limiting the local extinction of subordinate species through competitive exclusion (Schoener 1974) but also intraguild predation (Polis and Holt 1992; Lourenço and 2011; Garvey et al. 2022). Given the key role of predators in ecosystems, regulating food webs and resource distribution through top-down effects, studies comparing the diet of coexisting species are therefore highly needed, especially at a large geographical scale. In fact, this allows us to examine spatial variation according to environmental and climatic gradients, as well as assess the effects of variable interspecific competitive pressure.

Here, we focused on the niche partitioning and dietary differences among species belonging to the same foraging guild of medium-to-large-sized nocturnal raptors, feeding mostly on small mammals (Billerman et al. 2022; see also *Results*), across the Mediterranean Basin. More specifically, we examined diet differences in 3 owl species: the Northern long-eared owl (*Asio otus*; hereafter: long-eared owl), the tawny owl (*Strix aluco*), and the Eurasian eagle owl (*Bubo bubo*; hereafter: eagle owl). All these species mainly live in structured and mosaic habitats across the Palearctic (but the long-eared owl is also present in North America; Marks et al. 2020). On the one hand, the eagle owl mostly inhabits inaccessible areas with low human disturbance, but for some isolated individuals in urban settlements (Penteriani and Del Mar Delgado 2019; Billerman et al. 2022; but see Keller et al. 2020). On the other hand, the long-eared owl and the tawny owl are more habitat generalists throughout the year and are found mainly in heterogeneous, structured landscapes, but also in urban areas (Keller et al. 2020; Billerman et al. 2022). From a dietary point of view, the 3 species are considered to be specialists as mostly preying on small mammals and mostly hunt on the fly (Billerman et al. 2022). Therefore, the long-eared owl, tawny owl, and eagle owl constitute a foraging guild by sharing communal trophic resources and similar hunting strategies within comparable habitats (Billerman et al. 2022).

Studying the diet in this foraging guild is particularly eased by the great availability of pellets and subsequent identification of prey remains therein. In fact, owl pellets remain intact and reliable in providing estimates of mammal community features and diversity for a long time, with a higher likelihood of sampling rarer species as compared to traditional sampling methods (Terry 2010; Heisler et al. 2016). Therefore, a large number of studies reported the diet composition of owl species, providing a unique framework to address within-guild differences and diet variation at large spatial scales (Birrer 2009; Paniccia et al. 2018; Janžekovič and Klenovšek 2020; Romano et al. 2020, 2021; Riegert et al. 2021; Ratajc et al. 2023).

Considering the high similarity in diet, habitat selection and foraging strategies, competition among these 3 owl species is predicted to be high and mechanisms that enhance coexistence might have been favored by reducing the costs of competition. Specifically, we aimed at describing the species-specific diet features, while addressing within-guild differences in diet composition and diet variation according to geographical and environmental factors. We finally examined whether the diet of each species changed according to the presence or absence of the other(s). We focused on the Mediterranean Basin (Figure 1), one of the major global biodiversity hotspots in terms of species diversity and endemicity (Myers et al. 2000). It is also considered one of the main European Quaternary glacial refugia for mammals (southern refuge hypothesis: Sommer and Nadachowski 2006; Fløjgaard et al. 2009) as well as several other organisms (see review in Hewitt 1999). Moreover, the Mediterranean hosts several endemic species and a large proportion of the species of the Western Palearctic (Riegert et al. 2021), making it one of the most conservation-important areas for mammal fauna. Finally, the Mediterranean Basin is also a rather homogeneous bioclimatic region (Kottek et al. 2006), allowing us to compare populations that experience similar environmental conditions at a large scale.

**Figure 1.**
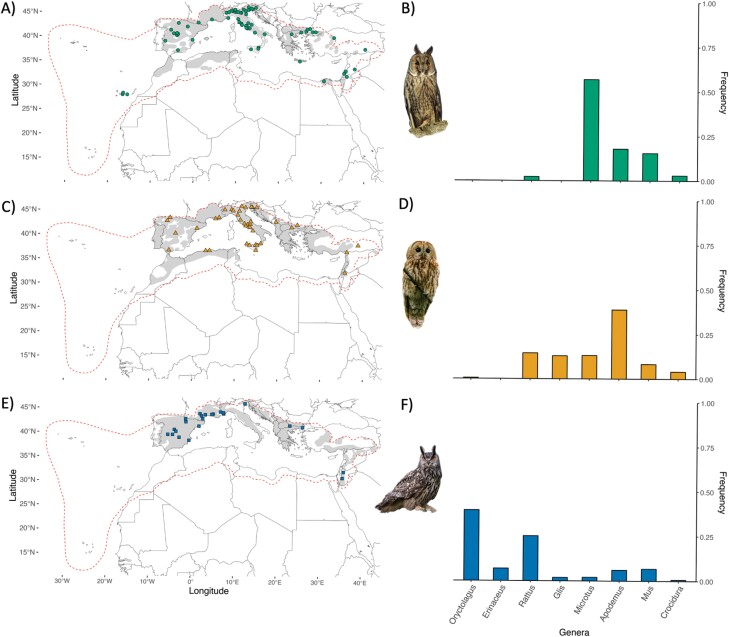
Distribution of the locations of diet data and frequency of the most common terrestrial mammals’ genera preyed by the (A, B) long-eared owl, (C, D) tawny owl, and (E, F) eagle owl in the Mediterranean Basin (dashed red line). Mammal’s genera are ordered by mean genera body size (see main text). Each species’ distribution range within the study area is shown in grey. Photo by E. Benussi, E. Crepet, and D. Panaretti.

## Materials and Methods

### Diet data and metrics

We gathered studies on diet variation in the long-eared owl, tawny owl, and eagle owl, which live in sympatry in the Mediterranean Basin. We excluded other similarly sized owl species living in the Mediterranean area because they strictly exploit agricultural landscapes (i.e., barn owl, *Tyto alba*), or their distribution range falls only marginally within the study area (i.e., Ural owl, *Strix uralensis* or short-eared owl, *Asio flammeus*), or their diet is mainly represented by birds (i.e., Tengmalm’s owl, *Aegolius funereus*) (Billerman et al. 2022). We considered as study area the Mediterranean Basin biodiversity hotspots’ limits provided by Hoffman et al. (2016), and included all the locations where the focal species are found within a 100 km buffer around them. In such a way, we obtained a larger spatial coverage, and we were thus able to better analyze the effect of both environmental and geographical variables on the 3 species’ diet (see also *Geographic and environmental diet variation*). We used a quasi-meta-analytical research framework, collecting studies from Web of Science, Google Scholar, Scopus, ResearchGate, Taylor and Francis online, Springer Link, and Google considering the following keywords “*Asio/A. otus*,” “long-eared owl,” “*Strix/S. aluco*,” “tawny owl,” “*Bubo/B. bubo,*” “eagle owl” combined with “pellet,” “diet,” “food,” “trophic,” “prey,” “foraging,” “Mediterranean.” We also included the names of all the countries in the Mediterranean Basin to improve the representativeness of under-sampled countries and searched through the reference list of each study and in journals’ archives using the same keywords. Overall, we gathered 160 papers reporting information on the diet of the long-eared owl (*N* = 90), tawny owl (*N* = 56), and eagle owl (*N* = 32). Some papers reported diet information for 2 (N = 14) or 3 (N = 2) of the predators here considered (see full list in Supplementary data).

We then associated each study with the location coordinates (latitude and longitude, expressed in decimal degrees), excluding studies addressing geographical scales larger than the used spatial resolution of diet data (i.e., spatial uncertainty larger than 20 km; see below) and those for which single precise locations could not be derived. To reduce the effect of spatial autocorrelation we pooled together locations closer than 20 km from each other (hereafter spatial clusters, see also *Statistical analyses*). In these cases, coordinates were obtained calculating the centroid of all the locations of a spatial cluster, and prey items were summed. Therefore, some datapoints contained information extracted from more than a single study. To obtain robust diet metric estimates, original data including diet information collected in multiple years and/or seasons in the same location were pooled and only locations with at least 90 terrestrial mammals prey items were considered (Romano et al. 2020, 2021). Whenever data were collected on islands we considered a threshold of 50 prey items (Romano et al. 2020). After these data selection procedures and geographical pooling, the final dataset comprised 128 datapoints (long-eared owl: *N* = 66; tawny owl: *N* = 39; eagle owl: *N* = 23).

For each datapoint, we computed diet metrics that reliably represent diet in terms of diversity, evenness, prey size, and proportion of terrestrial mammals. Following Romano et al. (2020), all diet metrics were calculated at the genus level and considering terrestrial mammals only. This was done because the majority of studies concentrate on terrestrial mammals only, as these account for the majority of prey items in all 3 species (Billerman et al. 2022; see also *Description of diet characteristics*), and to limit the bias due to different taxonomical detail in different studies. Whenever prey were reported at a higher taxonomical level than the genus (e.g., order), these were excluded from further analyses. We also excluded bat genera as these are only rarely found in pellets (Billerman et al. 2022) and pooled them in the category of other vertebrates (e.g., reptiles or birds; Romano et al. 2020). Invertebrates were not considered because they are only rarely reported in the studies because of their high rate of fragmentation in pellets (Fattorini et al. 2001) and, even when reported, account for a minor fraction of the total biomass of preys.

First, we calculated the Shannon diversity index: $H=\;-\sum p_{i}\ln p_{i}$, where p_i_ is the proportion of *i*^*th*^ genus, with higher values indicating a higher diversity (hereafter *H* index) (Shannon 1948). This metric, contrary to others, does not depend on the number of prey items, thus being particularly suitable to compare locations with different prey sample sizes (Romano et al. 2020). In fact, in all the 3 predator species there was no relationship between the *H* index and the number of prey items (i.e., terrestrial mammals) reported (linear models: long-eared owl: *t*_1, 63_ = −0.19, *P* = 0.85; tawny owl: *t*_1, 36_ = −0.64, *P* = 0.53; eagle owl: *t*_1, 18_ = 0.69, *P* = 0.50). We further calculated the Levins’ niche breadth index as $B=1/\sum p_{i}^{2}$ (Levins 1968), which was highly positively correlated with the *H* index in all the 3 predator species (Pearson’s correlation coefficient: long-eared owl, r = 0.95; tawny owl, r = 0.87; eagle owl, r = 0.91). Hence, we considered only the *H* index in further analyses. Second, we calculated Shannon evenness index: $J=\;\frac{H}{\ln G}$, where *H* is the *H* index and *G* is the number of genera (Pielou 1969). This index ranges between 0 and 1, with higher values representing a more equal representation of genera (hereafter *J* index). Third, we calculated the weighed mean prey size as the arithmetic mean body size of the *i*^*th*^ genus weighed for the number of prey items reported for the *i*^*th*^ genus (hereafter mean prey size). We obtained genera-specific body size estimates for mammals’ prey species from the EltonTraits database (Wilman et al. 2014), averaging body size data for all the species of the *i*^*th*^ genus that are found in the Mediterranean Basin countries (retrieved from the Map of Life www.mol.org in May 2022). Finally, we calculated the proportion of terrestrial mammal prey items over the total number of vertebrates recorded in the pellets. For this latter diet metric, we did not consider locations where mammals were the only considered taxon (i.e., papers omitting the information about the presence/absence of vertebrates other than mammals in the diet were excluded; *N* = 6).

### Geographic and environmental diet variation

We associated each location with both geographical and environmental variables averaged over a 20-km radius around each pair of coordinates. We note that variables averaged over a 50-km radius were strongly correlated to those averaged over a 20-km radius (Pearson’s correlation coefficient: elevation, *r *= 0.91; temperature, *r *= 0.93; precipitation, *r *= 0.99; tree cover, *r *= 0.88). Thus, we considered the 20-km radius, which better represents home ranges of all 3 species. For spatial clusters (i.e., whenever locations were pooled), geographical and environmental variables were first extracted for each location of the cluster and then averaged. Following Romano et al. (2020), we considered as geographical predictors latitude, longitude, elevation, and a two-level factor indicating whether the location was situated on the mainland (coded as 0) or on an island (coded as 1). This latter variable was only considered for the long-eared owl and the tawny owl because no diet data were available for the eagle owl on Mediterranean islands.

Then, we considered as environmental predictors the annual mean temperature, the annual cumulated precipitation, and the percentage of land covered by trees. These macroecological predictors are known to affect diet of nocturnal raptors (Rubolini et al. 2003; Penteriani and Del Mar Delgado 2019), as well as the distribution of both predators and small mammals (Fløjgaard et al. 2009; Brambilla et al. 2010; Jensen et al. 2012). We included temperature and precipitation at an annual resolution (mean and cumulated, respectively) instead of more temporally accurate ones (e.g., temperature of the warmest month) because of the large variability of temporal resolutions (i.e., from seasonal to decadal) addressed by the studies included in our database.

We obtained temperature and precipitation predictors from the CHELSA bioclimatic database (Karger et al. 2017), averaging variables between the years 1979 and 2013, and the tree land use cover from the CORINE Land Cover (European Space Agency 2019).

### Diet in sympatry versus allopatry

We also analyzed whether diet metrics for the long-eared owl and the tawny owl differed when in sympatry or allopatry with the other species. To this aim, each location where diet data were available was coded according to the presence or absence of the other species (0 = the other species of the foraging guild absent; 1 = the other species of the foraging guild present). We considered a species to be present (i.e., in sympatry) if the location coordinates were comprised within the other species’ distribution range (Supplementary Figure S1). Ranges were obtained from BirdLife International (http://datazone.birdlife.org/species/requestdis) and were cut on the study area (see *Diet data and metrics* and Figure 1). We conducted this analysis solely on the long-eared owl and the tawny owl because only 2 locations for the eagle owl did not overlap neither the tawny owl’s nor the long-eared owl’s range (i.e., competition factor = 0), therefore preventing us from performing statistical analyses.

### Statistical analysis

We analyzed dietary differences among the 3 owl species by first comparing diet metrics (*H* index, *J* index, mean prey size and proportion of terrestrial mammals) using ANOVAs and post hoc tests. We included diet metrics as dependent variables and the species identity as a categorical three-level factor. Then, whenever appropriate, we ran Tukey post hoc pairwise comparisons including a Bonferroni correction of the *P*-values to account for multiple testing. ANOVAs were run using the “car” R package (Fox and Weisberg 2019) and post hoc tests with the *glht* function of the “multcomp” R package (Hothorn et al. 2008).

We then analyzed spatial and environmental diet variation of each species according to geographical and environmental predictors using linear models fitted with the “glmmTMB” R package (Brooks et al. 2017). Linear models were run separately for the 3 species, fitting 2 sets of separated models for each species (i.e., *geographical* and *environmental* models). We considered diet metrics as dependent variables in all the models and either geographical (latitude, longitude, elevation, and island/mainland factor) or environmental (annual mean temperature, cumulated annual precipitation, and percentage of land covered by trees) predictors as independent variables. Due to precipitation and temperature being correlated for the eagle owl (r = −0.67), we only retained the latter in the linear models. In order to obtain scale-independent estimates, all continuous predictors were standardized within predator species. All diet metrics were analyzed using Gaussian distribution, but mean prey size and proportion of terrestrial mammals were first log_10_- and logit-transformed, respectively. We also tested in each model the occurrence of spatial autocorrelation using the Moran’s I index. We calculated this index over the residuals of each model and the inverse distance matrix of the locations’ coordinates using the *Moran.I* function of the “ape” R package (Paradis and Schliep 2019). We detected a significant spatial autocorrelation in 2 models (see Supplementary Tables S1–S2). In these models, we corrected for spatial autocorrelation by adding an exponential correlation structure that considered the pairwise distance matrix between all the pairs of locations’ coordinates (Romano et al. 2020).

We then compared diet variation across the Mediterranean Basin among the 3 species calculating the effect sizes for each diet metric and each model. Effect sizes *r* (partial correlation coefficient) were calculated using the formula $r=\;\frac{t}{\sqrt{t^{2}+df}}$, where *t* is the t-value attained by each given predictor in each linear model (i.e., separated by species) and *df* are the degrees of freedom obtained from the linear models. Then we obtained *Z*_*r*_, the Fisher transformation of the partial correlation coefficient, using the formula (Nakagawa and Cuthill 2007).

Finally, we analyzed the effect of competition on the diet metrics of the long-eared owl and the tawny owl. We considered each diet metric as a dependent variable and the competition factor (0 = the other species of the foraging guild absent; 1 = the other species of the foraging guild present) as an independent predictor in linear models. We fitted linear models separately for each species.

Model assumptions of normality of the residuals and heteroscedasticity were graphically checked with the “performance” R package (Lüdecke et al. 2021). All the models proved well, and no assumption was violated. All the analyses were run in R (R Core Team 2020; version 4.1.1).

## Results

### Description of diet characteristics

Overall, we relied on 229,381 prey items of which 212,236 were vertebrates (92.53%). Among vertebrates, 169,453 were mammals (79.84%), 35,679 were birds (16.81%), 4,352 were reptiles (2.05%), 2,290 were amphibians (1.08%), 455 were fishes (0.21%), and 7 were unidentified as amphibians/reptiles (<0.01%). Our database comprised 239 different vertebrate genera of which 139 were birds (58.16%), 66 mammals (27.62%), 16 reptiles (6.69%), 11 amphibians (4.60%), and 7 fishes (2.93%). Even if birds’ genera accounted for most of the genera reported, as compared to mammals, the number of prey items and the mean body size showed that mammals accounted for a much larger fraction of food biomass in all 3 species (long-eared owl: 78.20%; tawny owl: 68.34%; eagle owl 68.34%).

A general summary and a qualitative description of the diet metrics of the 3 species are reported in Table 1. In addition, to qualitatively compare the most preyed genera in the 3 species, we show the frequency of the 5 most preyed genera of mammal for each species (Figure 1). Three of these genera (*Apodemus, Mus,* and *Rattus*) were shared among all predators and one (*Microtus*) was common between long-eared owl and tawny owl, resulting in a total of 8 most commonly preyed genera. These 8 genera accounted on average for 86.19% of terrestrial mammals preyed genera (long-eared owl: 94.00%; tawny owl: 81.63%; eagle owl: 82.94%). The 3 predators differed in the most preferred preyed genus (long-eared owl: *Microtus;* tawny owl: *Apodemus*; and eagle owl: *Oryctolagus*). The eagle owl concentrated mostly on the biggest preys, while the long-eared owl and the tawny owl on the medium-small-sized mammals, also showing a higher overlap over the most commonly preyed genera (Figure 1). Even though the average body size of the *Apodemus* (27.3 g) is slightly lower than that of *Microtus* (31.7 g), the long-eared owl (i.e., the smallest predator) very rarely relied on bigger preys, apart from *Rattus*.

**Table 1.** Summary of quantitative diet characteristics (total number or mean ± SD among locations) and diet metrics (mean ± SD) of the long-eared owl, the tawny owl, and the eagle owl across the Mediterranean Basin

	Long-eared owl	Tawny owl	Eagle owl
Quantitative diet characteristics			
Number of locations	66	39	23
Number of preys	136,991	60,969	31,421
Number of vertebrate preys	133,756	47,586	30,894
Number of vertebrate genera	127	139	182
Number of terrestrial mammals genera	30	31	46
Mean number of vertebrate genera^a^	13.70 ± 9.80	18.62 ± 12.06	34.13 ± 25.40
Mean number of terrestrial mammals genera^a^	6.67 ± 2.59	9.46 ± 3.04	11.83 ± 6.12
Diet metrics			
*H* index	0.90 ± 0.34	1.30 ± 0.35	1.33 ± 0.47
*J* index	0.52 ± 0.17	0.63 ± 0.16	0.57 ± 0.19
Mean prey size (g)	44.01 ± 36.73	77.54 ± 85.93	917.27 ± 490.79
Percentage of terrestrial mammals (%)	82.78 ± 20.36	77.27 ± 23.70	69.66 ± 19.65

All diet metrics were calculated on terrestrial mammals only (see main text).

^a^Mean computed among locations.

### Comparison of diet metrics among owl species

**Figure 2.**
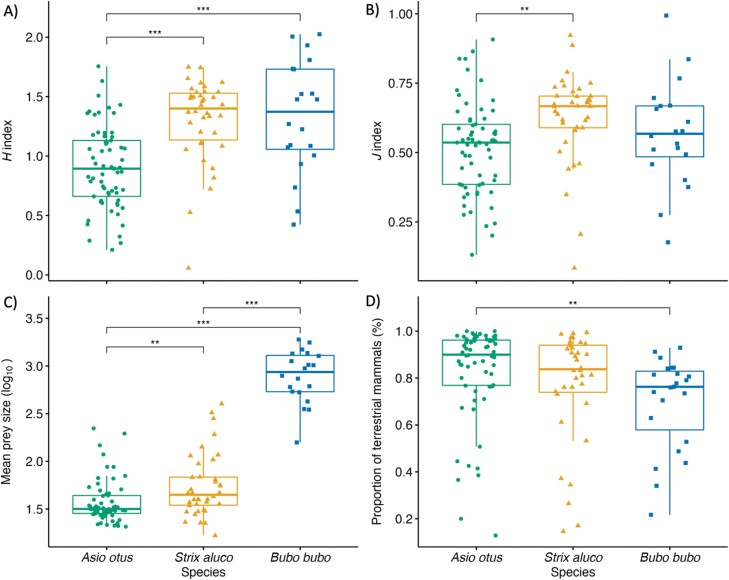
Differences in diet metrics (computed on terrestrial mammals only) among the long-eared owl, tawny owl, and eagle owl in the Mediterranean Basin. We show among-species differences in (A) *H* index, (B) *J* index, (C) mean prey size (expressed in g; log_10_-transformed), and (D) proportion of terrestrial mammals (logit-transformed). Significance of Tukey post hoc pairwise comparisons between species are indicated with stars (*P < 0.05, **P < 0.01, ***P < 0.001) and is calculated with *t*-tests with a Bonferroni correction for multiple testing.

We found significant differences in all diet metrics among species (*H* index: *F*_*2,120*_ = 19.05, *P* < 0.001; *J* index: *F*_*2,120*_* *= 5.11, *P *= 0.007; mean prey size: *F*_*2,121*_ = 192.3, *P *< 0.001; proportion of terrestrial mammals: *F*_*2,119*_* *= 5.34, *P* = 0.006). Prey diversity, evenness and mean prey size were lowest in the long-eared owl, which also showed the highest percentage of terrestrial mammals compared to the other species (Figure 2 and Supplementary Table S3). Hence, the long-eared owl was the most diverging species compared to the other predators, with most of the metrics being significantly different from both the tawny owl and the eagle owl (Figure 2 and Supplementary Table S3). In particular, *H* index and mean prey size were significantly lower than the other 2 species, while *J* index and proportion of mammals were, respectively, smaller than the tawny owl and larger than the eagle owl (Figure 2 and Supplementary Table S3). The tawny owl and eagle owl did not significantly differ in diet metrics, except for mean prey size, which is smaller in the former compared to the latter (Figure 2 and Supplementary Table S3). Finally, among-species differences in diet diversity and evenness did not qualitatively change when also accounting for birds as an additional single genus in the analyses (Supplementary Materials; Supplementary Figure S2).

### Geographic and environmental diet variation

**Figure 3.**
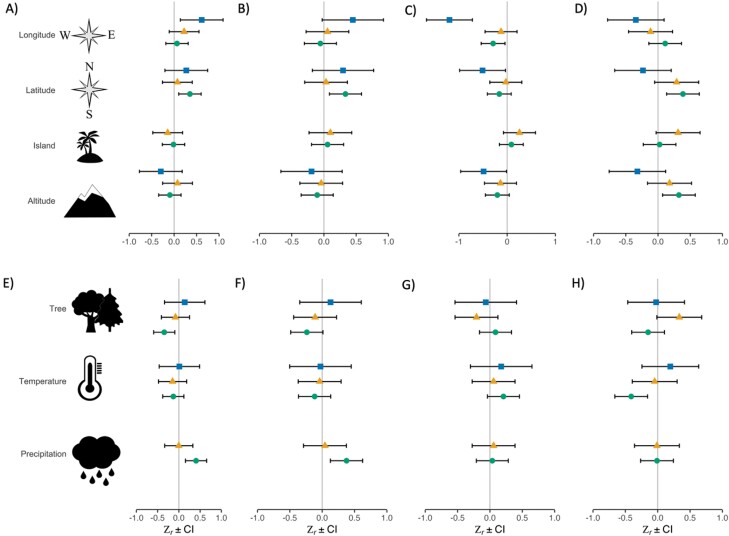
Forest plot showing Fisher transformation Z*r* effect sizes and 95% confidence intervals of the variation of diet metrics (computed on terrestrial mammals only) according to geographical (top row) and environmental predictors (bottom row). Variation of the (A, E) *H* index, (B, F) *J* index, (C, G) mean prey size, and (D, H) proportion of terrestrial mammals in the long-eared owl (green dots), tawny owl (yellow triangles), and eagle owl (blue squares) is shown.

Overall, we found that the 3 predators’ diets varied quite heterogeneously according to geographical and environmental predictors across the Mediterranean Basin (see Figure 3 and Supplementary Tables S4–S5). Diet diversity (*H* index) increased with longitude in the eagle owl and with latitude and cumulated precipitation in the long-eared owl, while decreased with tree cover in the long-eared owl. On the contrary, diet evenness (*J* index) was only influenced positively by latitude and negatively by the cumulated precipitation in the long-eared owl. The mean prey size decreased with longitude, latitude, and elevation in the eagle owl. Finally, the proportion of terrestrial mammals over vertebrates decreased with tree cover in the tawny owl and increased with mean annual temperature in the long-eared owl. All the other fixed effects were nonsignificant in all the species. Whenever a predictor variable significantly affected a diet metric (12 variables; Supplementary Tables S4–S5), in 7 cases, the direction of the relationship in the other 2 predator species was the same, while for the other 5 significant effects, the direction was different among the 3 species (see Figure 3, Supplementary Tables S4–S5). Such among-species heterogeneity in diet variation according to geographical and environmental predictors held true also when accounting for birds in the computation of both *H* and *J* indexes (Supplementary Materials; Supplementary Figure S3).

### Diet in sympatry versus allopatry

We found no significant difference between sympatric versus allopatric long-eared owls and tawny owls both in the mean prey size (long-eared owl: Estimate ± SE: −0.02 ± 0.07, *t*_1, 64_ = −0.30, *P* = 0.76; tawny owl: Estimate ± SE: 0.09 ± 0.11, *t*_1, 36_ = 0.79, *P* = 0.43) and in the proportion of terrestrial mammals (long-eared owl: Estimate ± SE: 0.27 ± 0.38, *t*_1, 59_ = 0.71, *P* = 0.48; tawny owl: Estimate ± SE: −0.43 ± 0.43, *t*_1, 33_ = −0.99, *P* = 0.32). On the contrary, we found a significant increase in both diet diversity (*H* index: Estimate ± SE: 0.24 ± 0.10, *t*_1, 63_ = 2.41, *P* = 0.016) and evenness (*J* index: Estimate ± SE: 0.13 ± 0.05, *t*_1, 63_ = 2.79, *P* = 0.005) in the long-eared owl when in sympatry with the tawny owl. However, a similar pattern was not found in the tawny owl, in which there was no significant difference in both the diet diversity (Estimate ± SE: −0.03 ± 0.12, *t*_1, 36_ = −0.30, *P* = 0.77) and evenness (Estimate ± SE: −0.05 ± 0.05, *t*_1, 36_ = −0.93, *P* = 0.35) when in sympatry or in allopatry with the long-eared owl. Moreover, considering that in sympatry there was a higher percentage of land covered by trees (logit-transformed) both for the long-eared owl (Estimate ± SE: 0.97 ± 0.35, *t*_1, 64_ = 2.77, *P* = 0.006) and the tawny owl (Estimate ± SE: 2.02 ± 0.88, *t*_1, 37_ = 2.30, *P* = 0.022), we also accounted for the effect of habitat by including the percentage of land covered by trees as an independent predictor in the linear models. This analysis confirmed the significant increase of both diet diversity (*H* index: Estimate ± SE: 0.29 ± 0.10, *t*_1, 62_ = 2.76, *P* = 0.006) and evenness (*J* index: Estimate ± SE: 0.14 ± 0.05, *t*_1, 62_ = 2.88, *P* = 0.004) for the long-eared owl but not for the tawny owl (*H* index: Estimate ± SE: −0.04 ± 0.12, *t*_1, 35_ = −0.33, *P* = 0.74; *J* index: Estimate ± SE: −0.05 ± 0.05, *t*_1, 35_ = −0.84, *P* = 0.40). All the relationships remained qualitatively unchanged when we also considered the eagle owl to define the competition factor (0 = the other species of the foraging guild absent; 1 = the other species of the foraging guild present; details not shown). Finally, considering birds in the diet diversity and evenness did not qualitatively change these results (Supplementary Materials). Allopatry locations were often found at the edge of each predator’s range (Supplementary Figure S1). Hence, we also run a supplementary randomization test to discriminate between the effect of the geographic position and the effect of being in sympatry versus allopatry. Such analysis showed that the differences in diet metrics are most likely due to being in sympatry versus allopatry rather than being explained by geographical position (see Supplementary Materials for further details).

## Discussion

Sympatric species belonging to the same foraging guild offer an ideal model to address dietary niche partitioning and study the mechanisms of ecological coexistence. Here, we investigated the differences in the dietary ecology of 3 species of a top-predator foraging guild (long-eared owl, tawny owl, and eagle owl), that live in sympatry across broad regions of the Mediterranean Basin. We found that the 3 species highly differed along niche axes, varying in terms of diet composition, diversity, evenness, mean prey size and proportion of terrestrial mammals. This is especially the case of the long-eared owl, which differed the most in diet features from the other predators. The 3 species’ diets were quite heterogeneously varying in response to geographical and environmental factors. Finally, we found an effect of being in sympatry versus allopatry with a significant increase in diet diversity and evenness in the long-eared owl when in sympatry with the tawny owl.

In accordance with previous studies, all 3 species mainly relied on small mammals (Birrer 2009; Penteriani and Del Mar Delgado 2019). The Mediterranean Basin has been a Pleistocene glacial refugium (Hewitt 1999), with a high speciation rate (Bilton et al. 1998) and is now particularly rich in small mammals. Hence, owl diet analyses in this biodiversity hotspot can aid research on mammal ecology and diversity across large spatial scales (Romano et al. 2020; Riegert et al. 2021), as it provides a straightforward and accurate method of sampling mammal communities (Heisler et al. 2016).

In accordance with dietary niche segregation, we showed a clear difference in the frequency of the most preferred genera, with *Microtus*, *Apodemus*, and *Oryctolagus* being the most preyed genera by the long-eared owl, tawny owl, and eagle owl, respectively. In this foraging guild, such dietary niche differences may be explained by several factors: habitat preferences, body size-driven prey selection, and competition avoidance. These 3 predators, even if at different degrees, exploit structured forests and natural areas (Billerman et al. 2022). Nonetheless, as previously shown in other taxa, coexisting sympatric species may reach dietary niche partitioning by segregating in different micro-habitats within the same landscape (Arlettaz et al. 1997), and consequently specializing over different prey taxa. The different proportions of *Microtus* voles and *Apodemus* mice—the most selected genera of the long-eared owl and the tawny owl, respectively—can thus be explained by spatial segregation within the same habitat. In fact, even though the 2 species may overlap in their foraging habitat, the tawny owl mostly hunts in wooded areas (Holt et al. 2020), where *Apodemus* mice are more abundant, whereas the long-eared owl also forages in open areas (Marks et al. 2020). Moreover, the latter is also known to forage during crepuscule with no strict restriction of foraging activity to night hours, as compared to the tawny owl. Hence, this activity pattern better matches the cathemeral activity of *Microtus* voles, while the tawny owl mainly relies on *Apodemus* mice which are, by contrast, strictly nocturnal (Halle and Stenseth 2000). Therefore, we might speculate that trophic niche divergence of these 2 species may be driven by an interplay of habitat and temporal segregation during foraging, which may have been adaptively selected as a mechanism to reduce trophic competition (Schoener 1974).

Contrary to previous studies on foraging niche divergence in morphologically equivalent species, for example, on bats (Arlettaz et al. 1997), here we addressed a foraging guild in which its element species are also morphologically different, with a clear variation in body size. Previous studies in owl species showed that dietary differences are related to body mass, with larger species preying on larger prey taxa (Comay and Dayan 2018). A similar pattern has also been found within lineage in the barn owl, with larger-bodied populations consuming a higher proportion of larger preys (Romano et al. 2021), suggesting that this pattern is coherent also at different taxonomical scales. Here, in accordance with previous studies, we showed that in 3 coexisting species, the eagle owl, the largest predator, forages on larger prey (i.e., mostly on *Oryctolagus* rabbits). Moreover, we also showed clear differences in diet metrics with the eagle owl being more generalist as compared to the smallest long-eared owl. In fact, the eagle owl attained the highest mammal prey diversity and at the same time the lowest proportion of terrestrial mammals. This also suggests a higher reliance on other prey taxa, especially birds, that may further broaden the diet of the eagle owl, as compared with the long-eared owl, which, in turn, has the highest proportion of terrestrial mammals. We may then argue that generalism versus specialism in this guild may be driven by the predator size and be a mechanism to avoid competitive exclusion (see e.g., Lanszki et al. 2019). In fact, it has been shown that the higher the difference in body size between 2 competitors, the lower the competition strength (Leyequién et al. 2007). Contrary to previous studies conducted in northern Europe (Nilsson 1984), but in accordance with those from the Mediterranean Levant (Comay and Dayan 2018), there was a clear difference between the mean prey size of the long-eared owl and the tawny owl. Nevertheless, the 2 species mostly preyed on similarly sized genera (long-eared owl: *Microtus* and tawny owl: *Apodemus*), but the long-eared owl very rarely preyed on larger preys, a process known as secondary resource partitioning (Gurd 2008). We may further argue that other factors, beyond segregation by size, may contribute to differences in prey size between the 2 species. For instance, we found a decrease in mean prey size according to increasing latitude coherent in all the 3 species (though this trend was statistically significant for the eagle owl only) as well as to decreasing temperature (coherent but not significant in any of the species). Even if this contradicts Bergmann’s rule, which postulates a decrease in body size with increasing temperature (Bergmann 1847), a similar pattern was also found in the barn owl on a global scale (Romano et al. 2020). This is known as converse Bergmann’s rule and was also reported for small mammal species (see e.g., Alhajeri and Steppan 2016; but see Ochocinska and Taylor 2003) and may thus explain such contrasting results. Studies addressing broader latitudinal ranges in these 3 species of predators may further shed light on the prey selection.

Diet metrics varied quite heterogeneously according to geographical and environmental factors in the 3 species. This was partly surprising as the Mediterranean is, at least climatically, quite homogeneous, and highlights the need for further studies to disentangle the complexity of interspecific diet variation and the effect of environmental conditions. In fact, niche theory predicts that coexisting species will partition trophic resources to prevent competitive exclusion (Schoener 1974). However, these predictions hold true until the environment is stable, and resources are limited. Environmental stochasticity (De León et al. 2014), climate change (Bond and Lavers 2014), and human-driven impacts (Elliott 2006) may exert a strong influence on dietary overlap, specialization, and strength of competition.

Finally, we found a significant increase in both diet diversity and evenness of the long-eared owl when in sympatry with the tawny owl, suggesting evidence of niche expansion. This was not the case for the tawny owl, whose diet metrics did not vary between sympatric and allopatric conditions with the long-eared owl. In these 2 species, it has already been shown that trophic competition may result in a reduction in the reproductive output of the long-eared owl, but not the tawny owl (Nilsson 1984). Moreover, there is evidence of tawny owls preying upon long-eared owls (Mikkola 1976). We may then argue that, when in sympatry with the tawny owl, the long-eared owl is subjected to both trophic competition and predation which may in turn cause an increase in dietary niche breadth and evenness, as a consequence of a lower reliance over the most preferred prey genera (i.e., *Apodemus*). We must note that these results might have been affected by the fact that most of the allopatry locations are situated at the borders of the study area, this being particularly true for the long-eared owl (Supplementary Figure S1). However, a randomization approach supported the interpretation that the differences in diet metrics are mainly due to the presence of a strong competition rather than an effect of taxonomically different communities located in different regions. This observation contrasts with previous reports in other regions: a study conducted in Sweden highlighted that the tawny owl broadened its diet when in the presence of the long-eared owl, but the contrary was not recorded (Nilsson 1984). We then advocate for more detailed field tests to better disentangle the effect of potential competitors on the top predators’ dietary ecology. Furthermore, we underline that the diet composition of each species strictly depends on the community structure, thus including not only other potentially competing species (in our case the Ural owl *Strix uralensis* or the Pharaoh eagle owl *Bubo ascalaphus*) but also predators and/or parasites. A deep community analysis may uncover more complex relationships as well as the effect of environmental factors and prey dynamics on the direction and strength of intraguild competition (Lourenço et al. 2011).

Moreover, we also documented intraguild predation (Lourenço and Rabaça 2006) with the eagle owl preying on the other 2 predators (51 prey remains of long-eared owls and 49 of tawny owls) and 4 cases of cannibalism in the eagle owl, imputable to preyed juvenile individuals (e.g., Rathgeber and Bayle 1997). Our results then highlight a quite complex food web for the 3 predator species, which may even have top-down effects over the entire community. We thus advocate for the use of both fine-grained Species Distribution Models and fine-scale behavioral studies. Such studies may offer a way to disentangle the complex patterns of both direct and indirect competition, tackling for instance the mechanisms through which the smaller predators, the so-called mesopredators (e.g., the long-eared owl), reduce interference competition and intraguild predation when in sympatry with their competitors (Garvey et al. 2022).

Overall, our results showed that 3 species belonging to the same foraging guild, and living in sympatry, had a segregation in foraging niche. Owl species differentiated diet in response to environmental factors and shifted prey when in sympatry. These findings suggest that dietary niche segregation in owl species, and more generally in foraging guilds, can lessen the disadvantages of competitive exclusion, in accordance with niche theory.

## Supplementary Material

Supplementary material can be found at https://academic.oup.com/cz.

zoae001_suppl_Supplementary_Material

zoae001_suppl_Supplementary_data

## Data Availability

Data and R scripts are available from the corresponding author upon reasonable request. The full list of papers included in the analyses is provided as supplementary data.
