# Supplementary material for: Trophic niche segregation in a guild of top predators within the Mediterranean Basin
Source: Curr Zool. 2024 Jan 30;70(6):697–706. doi: 10.1093/cz/zoae001 (PMC11634680; doi:10.1093/cz/zoae001)
Supplement: zoae001_suppl_Supplementary_Material [file zoae001_suppl_supplementary_material.docx]

SUPPLEMENTARY MATERIALS


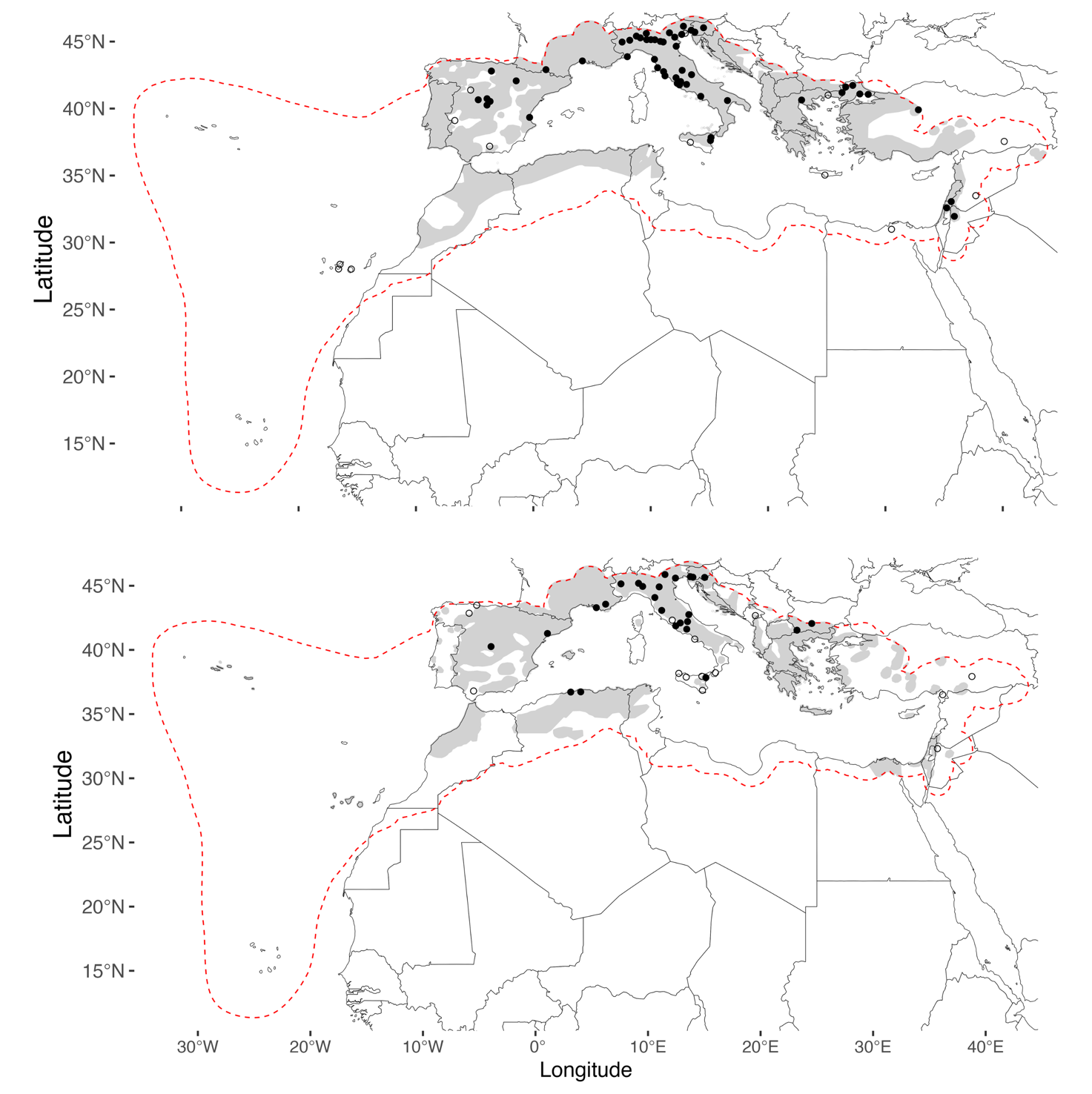


**Figure S1.** Sympatry between long-eared owl and tawny owl in the Mediterranean Basin (dashed red line). Locations of the long-eared owl (upper panel) and tawny owl (lower panel) are shown over the range of the other species (upper panel: tawny owl; lower panel: long-eared owl), highlighted in grey. Full dots represent locations of sympatry, i.e., where the other species is present (grey range).

**Table S1.** Moran’s I index for spatial autocorrelation calculated on the residuals of generalized linear models fitted for the diet metrics [*H* index, *J* index, mean prey size (log_10_-transformed), proportion of terrestrial mammals (logit-transformed)] according to geographical predictors. There was no model in which a significant spatial autocorrelation was detected.

| *H index* | | | |
| --- | --- | --- | --- |
|  | Moran’s I observed | Moran’s I expected | *P* |
| *Asio otus* | -0.04 | -0.02 | 0.59 |
| *Strix aluco* | -0.03 | -0.03 | 0.99 |
| *Bubo bubo* | -0.07 | -0.05 | 0.89 |
| *J index* | | | |
| *Asio otus* | -0.02 | -0.02 | 0.84 |
| *Strix aluco* | -0.03 | -0.03 | 0.91 |
| *Bubo bubo* | -0.23 | -0.05 | 0.08 |
| *Mean prey size* (log_10_-transformed) | | | |
| *Asio otus* | 0.03 | -0.02 | 0.51 |
| *Strix aluco* | -0.08 | -0.03 | 0.26 |
| *Bubo bubo* | 0.03 | -0.05 | 0.42 |
| *Proportion of terrestrial mammals* (logit-transformed) | | | |
| *Asio otus* | 0.01 | -0.02 | 0.95 |
| *Strix aluco* | -0.04 | -0.03 | 0.84 |
| *Bubo bubo* | -0.06 | -0.05 | 0.85 |

**Table S2.** Moran’s I index for spatial autocorrelation calculated on the residuals of generalized linear models fitted for the diet metrics [*H* index, *J* index, mean prey size (log_10_-transformed), proportion of terrestrial mammals (logit-transformed)] according to environmental predictors. Models in which a significant spatial autocorrelation was detected are highlighted in boldface.

| *H index* | | | |
| --- | --- | --- | --- |
|  | Moran’s I observed | Moran’s I expected | *P* |
| *Asio otus* | - 0.03 | - 0.02 | 0.77 |
| *Strix aluco* | - 0.01 | - 0.03 | 0.68 |
| *Bubo bubo* | 0.09 | - 0.05 | 0.15 |
| *J index* | | | |
| *Asio otus* | - 0.04 | - 0.02 | 0.53 |
| *Strix aluco* | - 0.04 | - 0.03 | 0.77 |
| *Bubo bubo* | - 0.06 | - 0.05 | 0.90 |
| *Mean prey size* (log_10_-transformed) | | | |
| ***Asio otus*** | **- 0.08** | **- 0.02** | **0.01** |
| *Strix aluco* | - 0.04 | - 0.03 | 0.73 |
| ***Bubo bubo*** | **0.36** | **- 0.05** | **< 0.001** |
| *Proportion of terrestrial mammals* (logit-transformed) | | | |
| *Asio otus* | - 0.04 | - 0.02 | 0.52 |
| *Strix aluco* | 0.05 | - 0.03 | 0.13 |
| *Bubo bubo* | 0.002 | - 0.05 | 0.59 |

**Table S3.** Tukey *post-hoc* comparisons (t-tests with a Bonferroni correction for multiple testing) of diet metrics [*H* index, *J* index, mean prey size (g, log_10_-transformed) and proportion of terrestrial mammals (logit-transformed)] among three sympatric owl species (long-eared owl, tawny owl and eagle owl) in the Mediterranean Basin. Statistically significant (*P* < 0.05) comparisons are highlighted in boldface.

|  |  | *H* index | | | |  | *J* index | | | |  | Mean prey size | | | |  | Proportion of terrestrial mammals | | | | |
| --- | --- | --- | --- | --- | --- | --- | --- | --- | --- | --- | --- | --- | --- | --- | --- | --- | --- | --- | --- | --- | --- |
|  |  | *ß* | SE | *t* | *P* |  | *ß* | SE | *t* | *P* |  | *ß* | SE | *t* | *P* |  | *ß* | SE | *t* | *P* |  |
|  | *A. otus – S. aluco* | **-0.40** | **0.08** | **-5.33** | **<0.001** |  | **-0.11** | **0.03** | **-3.18** | **0.006** |  | **-0.17** | **0.05** | **-3.09** | **0.007** |  | 0.37 | 0.25 | 1.48 | 0.43 |  |
|  | *A. otus – B. bubo* | **-0.43** | **0.09** | **-4.55** | **<0.001** |  | -0.05 | 0.04 | -1.19 | 0.71 |  | **-1.32** | **0.07** | **-19.43** | **<0.001** |  | **0.94** | **0.29** | **3.23** | **0.005** |  |
|  | *S. aluco – B. bubo* | -0.03 | 0.10 | -0.27 | 1 |  | 0.06 | 0.05 | 1.25 | 0.64 |  | **-1.15** | **0.07** | **-15.67** | **<0.001** |  | 0.57 | 0.32 | 1.80 | 0.23 |  |

**Table S4.** Linear models of diet metrics [*H* index, *J* index, mean prey size (g, log_10_-transformed) and proportion of terrestrial mammals (logit-transformed)] according to geographical predictors [latitude, longitude, elevation and a binary factor indicating whether the location was in the mainland (0) or on an island (1)]. Statistically significant effects (P < 0.05) are highlighted in boldface.

|  | | | *Asio otus* | | | | | | | *Strix aluco* | | | | | | | *Bubo bubo* | | | | |
| --- | --- | --- | --- | --- | --- | --- | --- | --- | --- | --- | --- | --- | --- | --- | --- | --- | --- | --- | --- | --- | --- |
| Predictors | | *ß* ± SE | | *t* | *P* | *r* | *Zr* ± *CI* |  | *ß* ± SE | | *t* | *P* | *r* | *Zr* ± *CI* |  | *ß* ± SE | | *t* | *P* | *r* | *Zr* ± *CI* |
| *H index* | | | | | | | | | | | | | | | | | | | | | |
|  | Latitude (°N) | **0.13 ± 0.05** | | **2.78** | **0.005** | **0.34** | **0.34 ± 0.25** |  | 0.03 ± 0.06 | | 0.41 | 0.68 | 0.07 | 0.07 ± 0.33 |  | 0.14 ± 0.12 | | 1.10 | 0.27 | 0.26 | 0.27 ± 0.48 |
|  | Longitude (°E) | 0.02 ± 0.04 | | 0.50 | 0.62 | 0.06 | 0.06 ± 0.25 |  | 0.07 ± 0.06 | | 1.29 | 0.20 | 0.22 | 0.22 ± 0.33 |  | **0.26 ± 0.10** | | **2.61** | **0.009** | **0.55** | **0.61 ± 0.48** |
|  | Elevation (m) | - 0.03 ± 0.04 | | - 0.74 | 0.46 | - 0.09 | - 0.09 ± 0.25 |  | 0.02 ± 0.06 | | 0.44 | 0.66 | 0.08 | 0.08 ± 0.33 |  | - 0.20 ± 0.17 | | - 1.20 | 0.23 | - 0.29 | - 0.30 ± 0.48 |
|  | Island/Continent (Island) | - 0.01 ± 0.13 | | - 0.11 | 0.91 | - 0.01 | - 0.01 ± 0.25 |  | - 0.15 ± 0.18 | | -0.83 | 0.41 | - 0.14 | -0.14 ± 0.33 |  | - | | - | - | - | - |
| *J index* | | | | | | | | | | | | | | | | | | | | | |
|  | Latitude (°N) | **0.06 ± 0.02** | | **2.64** | **0.008** | **0.32** | **0.33 ± 0.25** |  | 0.01 ± 0.03 | | 0.19 | 0.85 | 0.03 | 0.03 ± 0.33 |  | 0.07 ± 0.05 | | 1.20 | 0.23 | 0.29 | 0.30 ± 0.48 |
|  | Longitude (°E) | - 0.01 ± 0.02 | | - 0.43 | 0.67 | - 0.05 | - 0.05 ± 0.25 |  | 0.01 ± 0.03 | | 0.32 | 0.75 | 0.05 | 0.05 ± 0.33 |  | 0.08 ± 0.04 | | 1.85 | 0.06 | 0.42 | 0.45 ± 0.48 |
|  | Elevation (m) | - 0.02 ± 0.02 | | - 0.81 | 0.42 | - 0.10 | - 0.10 ± 0.25 |  | -0.01 ± 0.03 | | -0.24 | 0.81 | - 0.04 | - 0.04 ± 0.33 |  | - 0.06 ± 0.07 | | - 0.77 | 0.44 | - 0.19 | - 0.19 ± 0.48 |
|  | Island/Continent (Island) | 0.03 ± 0.06 | | 0.44 | 0.66 | 0.06 | 0.06 ± 0.25 |  | 0.05 ± 0.08 | | 0.58 | 0.56 | 0.10 | 0.10 ± 0.33 |  | - | | - | - | - | - |
| *Mean prey size (log_10_-transformed)* | | | | | | | | | | | | | | | | | | | | | |
|  | Latitude (°N) | - 0.04 ± 0.03 | | - 1.28 | 0.20 | - 0.16 | - 0.16 ± 0.25 |  | - 0.01 ± 0.06 | | - 0.16 | 0.88 | - 0.03 | - 0.03 ± 0.33 |  | **- 0.12 ± 0.05** | | **-2.13** | **0.033** | **- 0.47** | **- 0.51 ± 0.48** |
|  | Longitude (°E) | **- 0.06 ± 0.03** | | **- 2.31** | **0.021** | **- 0.28** | **- 0.29 ± 0.25** |  | - 0.04 ± 0.05 | | - 0.73 | 0.47 | - 0.13 | - 0.13 ± 0.33 |  | **- 0.26 ± 0.04** | | **-6.01** | **< 0.001** | **- 0.83** | **- 1.20 ± 0.48** |
|  | Elevation (m) | - 0.05 ± 0.03 | | - 1.60 | 0.11 | - 0.20 | - 0.20 ± 0.25 |  | - 0.04 ± 0.05 | | - 0.80 | 0.42 | - 0.14 | - 0.14 ± 0.33 |  | **- 0.15 ± 0.07** | | **-2.03** | **0.042** | **- 0.45** | **- 0.49 ± 0.48** |
|  | Island/Continent (Island) | 0.06 ± 0.08 | | 0.68 | 0.50 | 0.09 | 0.09 ± 0.25 |  | 0.24 ± 0.16 | | 1.48 | 0.14 | 0.25 | 0.26 ± 0.33 |  | - | | - | - | - | - |
| *Proportion of terrestrial mammals (logit-transformed)* | | | | | | | | | | | | | | | | | | | | | |
|  | Latitude (°N) | **0.51 ± 0.17** | | **3.02** | **0.003** | **0.37** | **0.37 ± 0.25** |  | 0.36 ± 0.22 | | 1.63 | 0.10 | 0.28 | 0.29 ± 0.34 |  | - 0.21 ± 0.20 | | - 1.03 | 0.31 | - 0.23 | - 0.23 ± 0.44 |
|  | Longitude (°E) | 0.13 ± 0.15 | | 0.85 | 0.40 | 0.11 | 0.11 ± 0.25 |  | - 0.13 ± 0.20 | | - 0.64 | 0.52 | - 0.11 | - 0.12 ± 0.34 |  | - 0.31 ± 0.20 | | - 1.53 | 0.13 | - 0.33 | - 0.34 ± 0.44 |
|  | Elevation(m) | **0.40 ± 0.16** | | **2.51** | **0.01** | **0.31** | **0.31 ± 0.25** |  | 0.20 ± 0.20 | | 1.00 | 0.32 | 0.18 | 0.18 ± 0.34 |  | - 0.25 ± 0.18 | | - 1.42 | 0.16 | - 0.32 | - 0.32 ± 0.44 |
|  | Island/Continent (Island) | 0.09 ± 0.46 | | 0.20 | 0.85 | 0.02 | 0.02 ± 0.25 |  | 1.11 ± 0.63 | | 1.76 | 0.08 | 0.30 | 0.31 ± 0.34 |  | - | | - | - | - | - |

**Table S5.** Linear models of diet metrics [*H* index, *J* index, mean prey size (g, log_10_-transformed) and proportion of terrestrial mammals (logit-transformed)] according to environmental predictors (mean annual temperature, annual precipitation amount and percentage of tree land use cover class). Statistically significant effects (*P* < 0.05) are highlighted in boldface.

|  | | *Asio otus* | | | | |  | *Strix aluco* | | | | | |  | | *Bubo bubo* | | | | | |
| --- | --- | --- | --- | --- | --- | --- | --- | --- | --- | --- | --- | --- | --- | --- | --- | --- | --- | --- | --- | --- | --- |
| Predictors | | *ß* ± SE | *t* | *P* | *r* | *Z_r_* ± *CI* |  | *ß* ± SE | *t* | *P* | *r* | *Z_r_* ± *CI* |  | | *ß* ± SE | | *t* | *P* | *r* | *Z_r_* ± *CI* | |
| *H index* | | | | | | | | | | | | | | | | | | | | | |
|  | Mean Temperature (° C) | - 0.05 ± 0.05 | - 1.02 | 0.30 | - 0.13 | - 0.13 ± 0.25 |  | - 0.07 ± 0.08 | -0.86 | 0.39 | - 0.15 | - 0.15 ± 0.33 |  | | 0.01 ± 0.27 | | 0.05 | 0.96 | 0.01 | 0.01 ± 0.48 | |
|  | Annual precipitation (mm) | **0.15 ± 0.05** | **3.25** | **0.001** | **0.38** | **0.40 ± 0.25** |  | - 0.001 ± 0.07 | -0.01 | 0.99 | - 0.002 | - 0.002 ± 0.33 |  | | - | | - | - | - | - | |
|  | Tree cover (%) | **- 0.13 ± 0.05** | **- 2.74** | **0.006** | **- 0.33** | **- 0.34 ± 0.25** |  | - 0.04 ± 0.08 | -0.47 | 0.64 | - 0.08 | - 0.08 ± 0.33 |  | | 0.09 ± 0.17 | | 0.57 | 0.57 | 0.14 | 0.14 ± 0.48 | |
| *J index* | | | | | | | | | | | | | | | | | | | | |  |
|  | Mean Temperature (° C) | - 0.02 ± 0.02 | - 0.91 | 0.36 | - 0.12 | - 0.12 ± 0.25 |  | -0.01 ± 0.04 | -0.23 | 0.82 | - 0.04 | -0.04 ± 0.33 |  | | -0.01 ± 0.11 | | -0.11 | 0.91 | -0.03 | -0.03 ± 0.48 | |
|  | Annual precipitation (mm) | **0.07 ± 0.02** | **3.01** | **0.003** | **0.36** | **0.38 ± 0.25** |  | 0.01 ± 0.03 | 0.25 | 0.80 | 0.04 | 0.04 ± 0.33 |  | | - | | - | - | - | - | |
|  | Tree cover (%) | - 0.04 ± 0.02 | - 1.87 | 0.06 | - 0.23 | - 0.24 ± 0.25 |  | -0.02 ± 0.04 | -0.64 | 0.52 | - 0.11 | -0.11 ± 0.33 |  | | 0.04 ± 0.07 | | 0.53 | 0.59 | 0.13 | 0.13 ± 0.48 | |
| *Mean prey size (log_10_-transformed)* | | | | | | | | | | | | | | | | | | | | |  |
|  | Mean Temperature (° C) | 0.06 ± 0.04 | 1.65 | 0.10 | 0.21 | 0.21 ± 0.25 |  | 0.02 ± 0.07 | 0.33 | 0.74 | 0.06 | 0.06 ± 0.33 |  | | 0.08 ± 0.10 | | 0.72 | 0.47 | 0.17 | 0.17 ± 0.48 | |
|  | Annual precipitation (mm) | 0.01 ± 0.04 | 0.29 | 0.77 | 0.04 | 0.04 ± 0.25 |  | 0.02 ± 0.07 | 0.33 | 0.74 | 0.06 | 0.06 ± 0.33 |  | | - | | - | - | - | - | |
|  | Tree cover (%) | 0.02 ± 0.04 | 0.68 | 0.50 | 0.09 | 0.09 ± 0.25 |  | -0.09 ± 0.07 | -1.22 | 0.22 | - 0.20 | - 0.21 ± 0.33 |  | | -0.01 ± 0.04 | | -0.27 | 0.79 | -0.06 | -0.06 ± 0.48 | |
| *Proportion of terrestrial mammals (logit-transformed)* | | | | | | | | | | | | | | | | | | | | |  |
|  | Mean Temperature (° C) | **- 0.60 ± 0.18** | **- 3.23** | **0.001** | **-0.39** | **-0.41 ± 0.25** |  | -0.07 ± 0.31 | -0.26 | 0.79 | - 0.04 | - 0.04 ± 0.34 |  | | 0.22 ± 0.26 | | 0.86 | 0.39 | 0.19 | 0.19 ± 0.44 | |
|  | Annual precipitation (mm) | - 0.01 ± 0.17 | - 0.08 | 0.94 | -0.01 | -0.01 ± 0.25 |  | -0.02 ± 0.32 | -0.07 | 0.94 | - 0.01 | - 0.01 ± 0.34 |  | | - | | - | - | - | - | |
|  | Tree cover (%) | - 0.21 ± 0.18 | - 1.14 | 0.25 | -0.15 | -0.15 ± 0.25 |  | 0.59 ± 0.31 | 1.92 | 0.054 | 0.32 | 0.33 ± 0.34 |  | | -0.03 ± 0.26 | | -0.11 | 0.92 | -0.02 | -0.02 ± 0.44 | |

**Supplementary analyses: Birds in the diet**

We repeated the diet metrics analyses and included birds as a single genus along with all the terrestrial mammals (see Main text). We could not reliably use all the genera reported for birds as different studies reported birds at different taxonomical detail (e.g., some as orders, some as families). We only included locations with at least 90 or 50 prey items for continental and island locations, respectively (see also Main text). The number of locations for which we had data for both birds and mammals is 129 (long-eared owl: 68; tawny owl: 35; eagle owl: 26). For these, we then cumulated all the birds in a single genus and used this in the computation of diet metrics of both diversity (*H* index) and evenness (*J* index). We analysed among-species differences with ANOVAs and post-hoc tests, diet variation and the effect of competition with linear models (see Main text for further details).

Among-species differences in diet diversity and evenness were qualitatively similar to what we found including only terrestrial mammals (see Fig. 2). In particular, we found significant differences in terms of both diversity (*H* index: *F_2,128_* = 21.49, *P* < 0.001) and evenness (*J* index: *F_2,127_* = 7.24, *P* = 0.001) (Fig. S2). As we found in the analyses including only terrestrial mammals (see Main text), the long-eared owl was the most diverging predator species. In fact, it attained the lowest diet diversity and evenness, as compared to the other two predators (Fig. S2). The eagle owl had the highest average diet diversity, confirming what we found with terrestrial mammals.


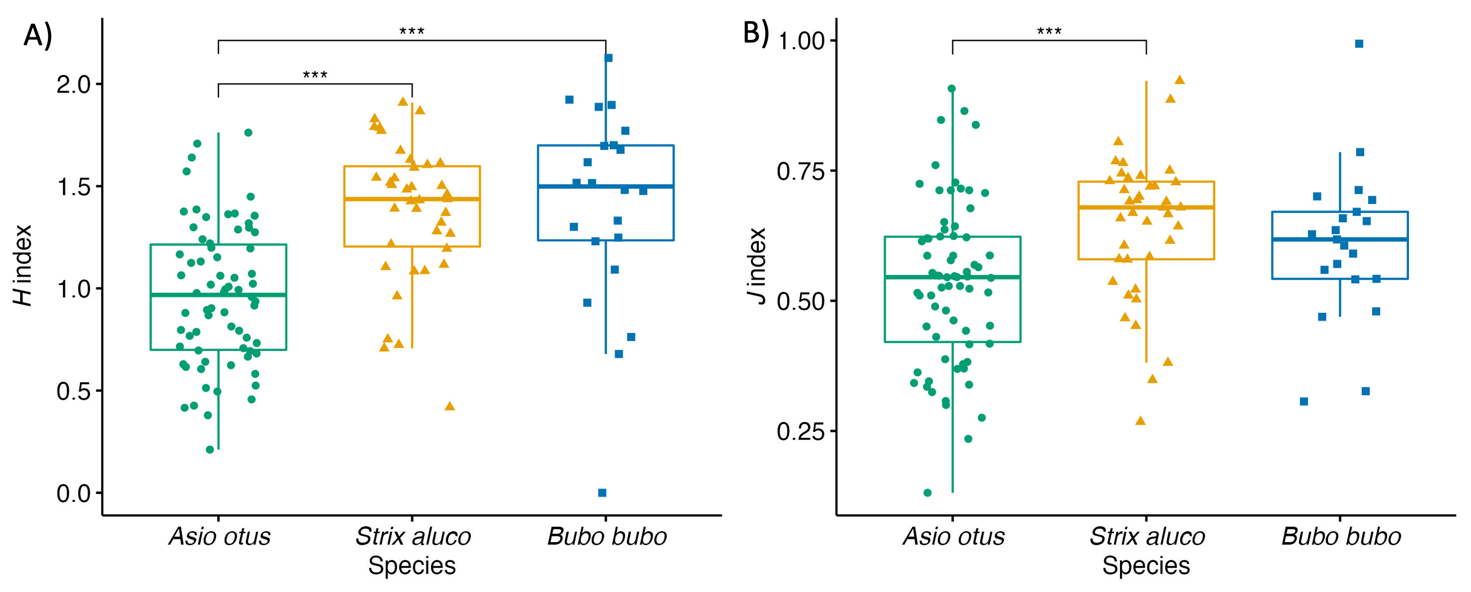


**Figure S2.** Differences in diet metrics (computed on terrestrial mammals and birds) among the long-eared owl, tawny owl and eagle owl in the Mediterranean Basin. We show among-species differences in A) *H* index and B) *J* index. Significance of Tukey *post-hoc* pairwise comparisons between species are indicated with stars (**P <* 0.05, ** *P* < 0.01, *** *P* < 0.001) and is calculated with t-tests with a Bonferroni correction for multiple testing.

We then built linear models with both geographical and environmental predictors analysing the variation of both diet diversity (*H* index) and evenness (*J* index). The direction of predictors remained qualitatively similar to what we found when considering terrestrial mammals only, as the comparison between Fig. 3 and Fig. S3 shows. This confirms the high among-species heterogeneity we found in the effect of environmental variables on diet metrics (see also Main text).


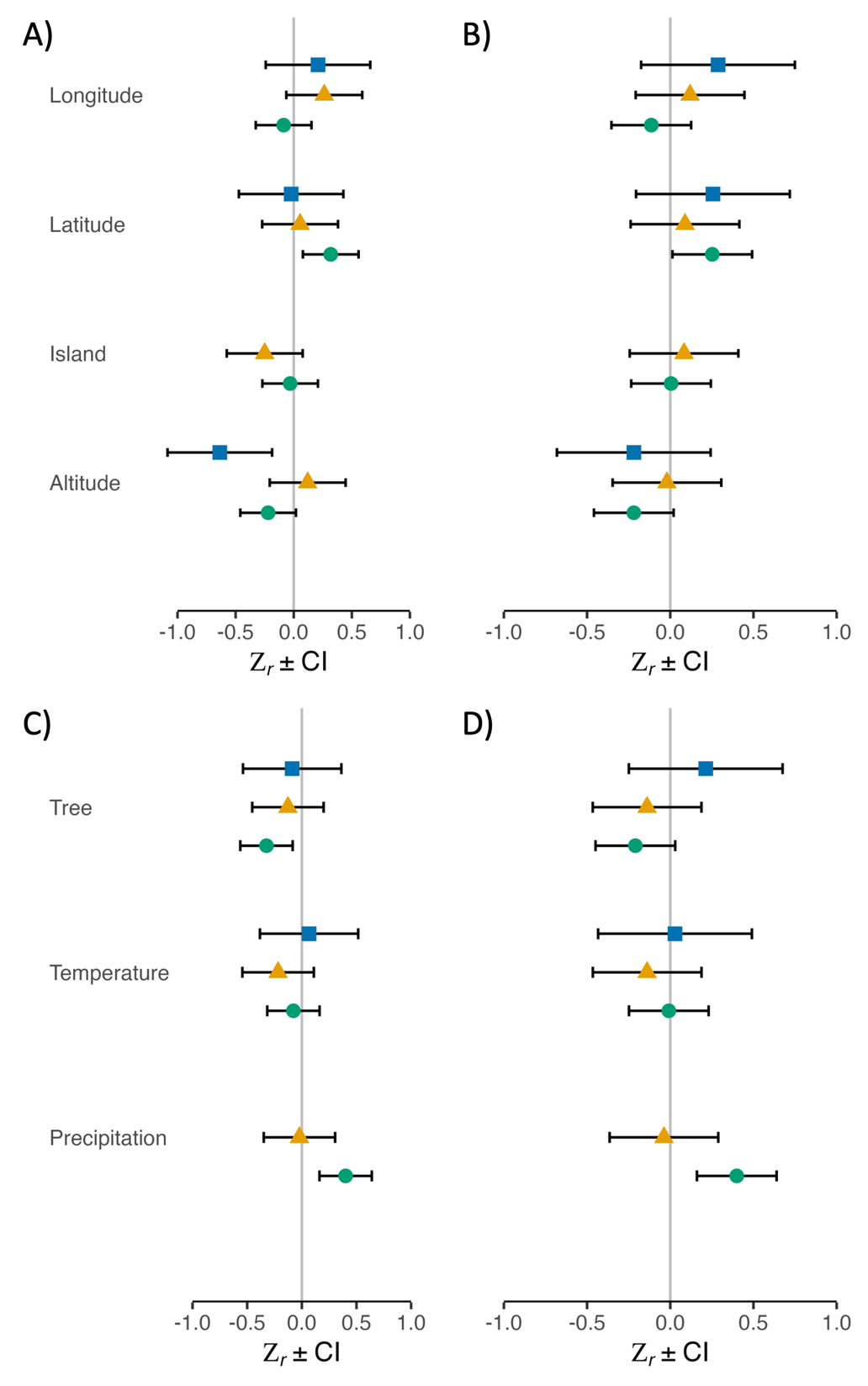


**Figure S3.** Forest plot showing Fisher transformation Z*r* effect sizes and 95% Confidence Intervals (CI) of the variation of diet metrics according to geographical (top row) and environmental predictors (bottom row). Variation of the (A,C) *H* index, and (B, D) *J* index in the long-eared owl (green dots), tawny owl (yellow triangles) and eagle owl (blue squares) is shown.

Finally, we analysed the effect of competition (sympatry vs allopatry) on both diet diversity and evenness in the long-eared owl and tawny owl (see Main text for further details). The results did not qualitatively change, with both an increase in diet diversity (Estimate ± SE: 0.20 ± 0.09, *t*_1, 63_ = 2.13, *P* = 0.033) and evenness (Estimate ± SE: 0.09 ± 0.04, *t*_1, 63_ = 2.11, *P* = 0.034) in the long-eared owl when in sympatry with the tawny owl. We did not find such pattern in the tawny owl’s diet diversity (Estimate ± SE: -0.003 ± 0.11, *t*_1, 36_ = -0.03, *P* = 0.98) and evenness (Estimate ± SE: -0.02 ± 0.05, *t*_1, 36_ = -0.53, *P* = 0.59).

**Supplementary analyses: sympatry vs. allopatry**

Allopatry diet locations were often found at the borders of the study area, which also corresponds to the geographical limits of the species’ distribution ranges (see also Fig. S1). Hence, we also run a supplementary analysis to ensure that the differences we found between allopatry and sympatry (see Main text) were not actually due to geographical differences in prey communities between the centre and peripheral distribution range of the predators.

To obtain an “allopatry-sympatry” vs “sympatry” comparison, we created two different groups: 1) we merged the allopatry locations with their nearest neighbours in sympatry to obtain “allopatry-sympatry” locations. Then 2) we randomly paired two sympatry locations to obtain “sympatry” locations. Hence, on the one hand, we obtained one group of allopatry-sympatry locations (one allopatry point and its nearest neighbour; N = 27). On the other hand, we created 1000 sets of “sympatry” locations (N = 22) resulting from the sympatry pairs.

For each of these new locations, we computed the diet diversity and evenness aggregating all the preys of the two locations of the pair. With these, we fitted 1000 linear models including the diet metrics as a response variable and a 2-level factor indicating whether it was in the allopatry-sympatry or sympatry group. In other words, we compared allopatry-sympatry (allopatry and nearest neighbour) and sympatry diet metrics.
In the long-eared owl, in all the 1000 linear models, the diet diversity was lower in the allopatry-sympatry cohorts (mean Z = - 1.06; min. = - 1.86; max. = - 0.33) but this difference was never statistically significant (mean P-value = 0.31; min. = 0.06; max = 0.74). The same also applied to the evenness, even though some models indicated that the evenness was higher in the allopatry-sympatry cohorts (mean Z = - 0.68; min. = - 1.74; max. = 0.34). Nevertheless, this difference was again never statistically significant (mean P-value = 0.52; min. = 0.08; max = 0.99).

We run the same analysis on the tawny owl as well. Similarly to the long-eared owl, the 1000 linear models showed no significant difference between allopatry-sympatry and sympatry cohorts both for diet diversity (mean P-value = 0.56; min. = 0.15; max = 0.91) and diet evenness (mean P-value = 0.76; min. = 0.22; max = 0.99). In this species, the diet diversity was always lower in allopatry-sympatry cohorts (mean Z = - 0.62; min. = - 1.44; max. = 0.12). However, this was not the case for the diet evenness, for which some models indicated a higher value in allopatry-sympatry cohorts (mean Z = - 0.09; min. = - 1.20; max. = 0.83).

In other words, we found that diet diversity and evenness did not statistically differ between allopatry-sympatry and sympatry locations. Hence, we conclude that the differences we observed in the main analyses are most likely due to the locations being in allopatry vs. sympatry rather than being due to geographical differences between the periphery and the centre of the predator’s distribution range.
